# Optical Sensors Based on Whispering Gallery Modes in Fluorescent Microbeads: Response to Specific Interactions

**DOI:** 10.3390/s100606257

**Published:** 2010-06-22

**Authors:** Michael Himmelhaus, Sivashankar Krishnamoorthy, Alexandre Francois

**Affiliations:** 1 Fujirebio, Inc., 51 Komiya-cho, Hachioji-shi 192-0031, Tokyo, Japan; 2 nanoBioAnalytics, Schwarzschildstr. 8, 12489 Berlin, Germany; 3 Institute of Materials Research and Engineering, 3, Research Link, 117602, Singapore; E-Mail: krishnamoorthys@imre.a-star.edu.sg; 4 Centre of Expertise in Photonics, School of Chemistry and Physics, The University of Adelaide, SA5005, Australia; E-Mail: alexandre.francois@adelaide.edu.au

**Keywords:** whispering gallery modes, cavity modes, optical sensing, refractive index sensing, biosensing, label-free detection

## Abstract

Whispering gallery modes (WGMs) in surface-fixated fluorescent polystyrene microbeads are studied in view of their capability of sensing the formation of biochemical adsorption layers on their outer surface with the well-established biotin-streptavidin specific binding as the model system. Three different methods for analysis of the observed shifts in the WGM wavelength positions are applied and used to quantify the adsorbed mass densities, which are then compared with the results of a comparative surface plasmon resonance (SPR) study.

## 1. Introduction

Label-free bioanalytical systems and immunodiagnostics devices require a transducer mechanism that translates a specific binding event into a physical and eventually electronic signal, which can be further processed. The most successfully applied transducer mechanisms so far are either sensitive to the mass or the dielectric properties of the bound material, such as electric field effect transistors, quartz microbalance and acoustic wave sensors, microcantilevers, fiber sensors and optical waveguides, and systems based on either localized or propagating surface plasmons [1].

**Figure 1.**
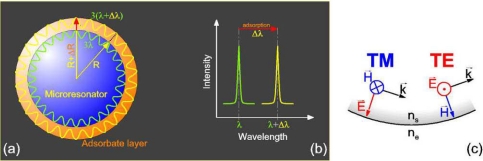
Illustration of the origin of cavity mode shifts in a WGM resonator upon biomolecular adsorption: **(a)** Effect of resonator size increase due to an adsorbate layer on whispering gallery mode (WGM) formation; **(b)** WGM wavelength shift due to formation of the adsorbate layer; **(c)** Definition of the two states of polarization for WGMs: *k⃗*—wave vector, *E⃗*—electric field, *H⃗*—magnetic field, n_s_—refractive index of the resonator, n_e_—refractive index of the environment.

Recently, a new class of label-free optical sensors has been introduced, which can be regarded as microscopic closed-loop waveguide sensors [2,3]. The principle of operation, which is depicted in Figure 1, is based on the entrapment of light inside of a small dielectric sphere by total internal reflection, where it recirculates in an arbitrary plane of propagation and steadily probes the ambient of the sphere along its way by an evanescent field, which extents typically for few hundreds of nanometers into the sphere’s environment. In contrast to an “open-loop” evanescent field sensor that applies freely propagating light rays, such as most fiber and waveguide sensors, the sphere acts as a spherical optical cavity, in which only certain optical modes, the so-called “whispering gallery modes” (WGMs), are allowed due to self-interference of the recirculating rays [4]. Obviously, this resonator condition is not only sensitive to the sphere’s dielectric environment but also depends on the sphere size, thereby introducing an additional component into the transducer mechanism. When, as typical for on-chip biosensors, an adsorption layer forms on the sphere surface (*cf.,* Figure 1a), not only the dielectric properties within the evanescent field of the propagating waves will change, but also the resonator condition due to the size increase ΔR of the sphere with initial radius R. Accordingly, as sketched in Figure 1b, the formation of an adsorption layer will be observed as a shift in the WGM positions towards higher wavelengths with a magnitude of Δλ/λ ∝ ΔR/R. This effect should kick in particularly on microscopic scale, where the thickness of a typical biomolecular adsorption layer is no longer negligible with respect to the sphere size.

The first works exploiting this transducer principle for optical sensing embodied silica spheres of some hundreds of micrometers in diameter and applied an evanescent field coupling scheme for WGM excitation [2,3]. The advantage of this approach is related to the extreme high quality (Q-) factors that can be achieved with silica spheres in this size regime [5], which in turn yields extremely narrow bandwidths of the optical modes and thus very high sensitivity for the detection of alterations of both mode positions [3] and bandwidths [2].

Recently, WGM-based sensors in much smaller particles with sizes from 2–15 μm have also been explored for their applicability to refractive index [6, 7, 8] and biosensing [9, 10]. In contrast to above-mentioned silica spheres, these sensors are operated in the “low-Q” regime, *i.e.*, their WGMs exhibit already significant bandwidths typically in the range of 0.02–0.2 nm. This drawback in resolution, however, can be compensated by the larger shift Δλ ∝ λ ΔR/R of the mode positions in smaller spheres upon adlayer formation [9]. Further, also the free spectral range of the resonator, *δλ* ∝ *λ*^2^ / *R*, increases with decreasing sphere size, thereby reducing the spectral mode density as compared to high-Q sensors. This enables the detection of a group of individual modes by means of a spectroscopic system. The simultaneous determination of more than a single mode position is advantageous, since the mode spacing contains information about the resonator dimension.

In a recent study on the applicability of WGM sensors to refractive index sensing [8], we utilized spectra of low-Q WGM sensors for simultaneous determination of environmental refractive indices and sphere sizes, which both are also crucial parameters with regard to biosensing applications. In the following, we will explore whether and if, to what extent, the same evaluation scheme can be applied to biosensing. The results are compared to those obtained by means of a simpler scheme based on ray optics and an analytical approach based on perturbation theory [11]. On the experimental side, surface plasmon resonance (SPR) is applied to put the results achieved into a broader context of state-of-the-art label-free biosensing.

## 2. Theory

The full treatment of WGMs in spherical cavities within the framework of classical electromagnetism requires the application of Mie/Debye theory, which is quite demanding in terms of the computational effort involved due to the need for numerical calculation of the sums of series of Bessel functions [12]. This can be avoided with simpler models based, for example, on elementary optics or on analytical approximations to the exact Mie/Debye solutions. In the following, we will introduce two of such models, one of which applies an elementary ray optics approach, while the second one takes advantage of the analytical formulation of Airy approximations to the full Mie/Debye solutions [7,13].

### 2.1. Ray Optics Model

The simplest and most illustrative description of mode positions in a spherical cavity is directly deduced from the cyclic boundary condition of a light ray recirculating in a plane of propagation inside of a spherical cavity (*cf.,* Figure 1a):

(1) $$\lambda_{m}=\frac{2\;\pi n_{s}\;R}{m}$$

Here, *λ_m_* is the vacuum wavelength of the WGM with the integer mode number *m*, *n_s_* the refractive index of the sphere, and *R* its radius. The mode number can be interpreted as the number of full wavelengths *λ_m_* that fit into the sphere’s circumference with the optical path length *2 πn_s_R*. Equation 1 basically states that the ray has to return in phase to a point where it originally started.

A similar result can be obtained also when combining a ray optics approach with the photon picture [14]. A photon with linear momentum |*p⃗*| = *ħ k* = 2 *π n_s_ ħ/λ* circulating at a distance *R* from the center of a sphere with index *n_s_* has an angular momentum of |*L⃗*| = *R* |*p⃗*| = 2 *π n_s_ R ħ/λ*. At the same time, according to Mie/Debye theory the angular momentum of a WGM with mode number *m* is given by $\left|\vec{L}\right|=\sqrt{L^{2}}=\sqrt{m(m+1)\hbar^{2}}\approx (m+\frac{1}{2})\hbar$. Equating these two relations for |*L⃗*| yields $\lambda_{m}=\frac{2\;\pi \;n_{s}\;R}{m+\frac{1}{2}}$ [15]. The additional term ½ corresponds to an additional phase shift of the recirculating photon, which accounts for the fact that strictly speaking, after one full roundtrip, the ray hits the point of its origin at a different angle from that under which it emerged. For large *m*, *i.e*., not too small cavities, this difference can be neglected, *i.e.,* the emerging and recirculating beams are basically collinear.

One of the advantages of Equation 1 is that the thickness of a layer of refractive index identical to that of the sphere adsorbed on the sphere surface can be directly determined once the mode shift *Δλ_m_* has been measured. As illustrated in Figure 1, an adsorbate layer increases the sphere size by an increase in its radius, *ΔR*, and thus causes a mode to increase its wavelength from *λ_m_* to *λ′_m_* > λ*_m_* to account for the increase of the circumference while keeping the number of wavelengths fitting into one roundtrip, *i.e.*, the mode number *m*, constant. Therefore, with *m′* = *m* from Equation 1 follows:

(2) $$\begin{array}{ll} {\Delta \lambda }_{m} & =\lambda_{m}^{\prime }-\lambda_{m}=\frac{2\;\pi \;n_{s}\;(R+\Delta R)\;}{m}\;\;-\;\;\frac{2\;\pi \;n_{s}\;R\;}{m}=\frac{2\;\pi \;n_{s}\;\Delta R\;}{m}\\ & =\lambda_{m}\frac{\Delta R}{R} \end{array}$$

From thus obtained change in radius *ΔR*, the volume change *ΔV* can be calculated and from this value, the known mass density of the adsorbate *ρ*, and the surface area of the sphere *A*, the surface mass density *σ* can be obtained according to:

(3) $$\sigma =\rho \frac{\Delta V}{A}=\rho \frac{\frac{4}{3}\;\pi \;\left[{\left(R+\Delta R\right)}^{3}-R^{3}\right]}{4\;\pi \;R^{2}}=\frac{\rho }{3}\frac{{\left(R+\Delta R\right)}^{3}-R^{3}}{R^{2}}$$

Obviously, in practice the change in sphere size *ΔR* as given by Equation 2 must be interpreted as an effective increase, thereby neglecting the different dielectric constants of sphere and adsorbate. This is for most proteins with a refractive index of about 1.5 and most cavity materials with refractive indices from about 1.44 (for silica) to 1.59 (for polystyrene) a reasonable assumption. Moreover, we found recently that the refractive index of dye-doped polystyrene (PS) spheres is lower than its literature value and typically lies in the range of 1.54–1.55 [8].

It is somewhat surprising that equations 1 and 2 neither account for the polarization of the WGMs (transverse magnetic, TM, or transverse electric, TE; for definition *cf.,* Figure 1c) nor for the refractive index of the sphere’s environment, which both seem to be crucial parameters for the WGM positions. The reason for this lack is related to the evanescent-field nature of WGMs. For total internal reflection at a dielectric/dielectric interface it is well known that the evanescent field propagates along the boundary with the phase velocity of the denser medium, although it reaches out into the less dense medium. Also, the polarization dependence of the evanescent fields, e.g., in terms of their decay lengths, is small and thus is neglected in equations 1 and 2. Nevertheless, it should be kept in mind that such dependency exists. Therefore, and because of above-mentioned approximation with regard to the adsorbate index, in the following we will denote radii calculated from the experimental mode spacings of TM and TE modes, respectively, by means of equations 1 and 2 with *R^TM^* and *R^TE^*, respectively, to indicate their dependency on these conditions and to distinguish them from the geometrical radius *R* of the sensor.

### 2.2. Airy Approximations

The basic problem of calculating the eigenmodes of a dielectric sphere with index *n_s_* embedded in a dielectric medium with index *n_e_* has been solved within the framework of Maxwell’s electromagnetic theory already hundred years ago by Mie and Debye. Unfortunate from a practical viewpoint is, however, that the solutions are presented in form of infinite series of Bessel functions of all three different kinds, which implies not only a good deal of computational effort, but also raises the question where to truncate analytically infinite series in practice [12].

For these reasons, Airy approximations have been derived [13], which can be presented in analytical form and which describe the mode positions well within an error of *ν*^−1^, where *ν* is the order of the Bessel function involved and related to the mode number *m* of the corresponding WGM by *ν* = *m* + ½.

On this basis, Pang *et al*. [7] recently deduced formulas for WGM mode positions for the general case of an environmental index n_e_ ≠ 1 as given by:

(4a) $$\lambda_{\mathrm{TM}}(q=1,m,R,n_{s},n)=2\pi n_{s}\;R\left(\nu +1.8557\;\;\nu^{1/3}-\frac{1}{n\sqrt{n^{2}-1}}+1.0331\;\;\nu^{-1/3}-\frac{1.8557(n^{4}-\frac{2}{3})}{n^{3}{\left(n^{2}-1\right)}^{3/2}}\nu^{-2/3}+O(\nu^{-1})\right)$$

(4b) $$\lambda_{\mathrm{TE}}(q=1,m,R,n_{s},n)=2\pi n_{s}\;R{\left(\nu +1.8557\;\;\nu^{1/3}-\frac{n}{\sqrt{n^{2}-1}}+1.0331\;\;\nu^{-1/3}-\frac{0.6186\;\;n^{3}}{{\left(n^{2}-1\right)}^{3/2}}\nu^{-2/3}+O(\nu^{-1})\right)}^{-1}$$

Here, *λ_TM_* and *λ_TE_* describe the wavelength positions of first order (*i.e., q* = 1) TM and TE modes with mode number *m*, *R* the sphere’s geometrical radius, and *n* = *n_s_*/*n_e_* the refractive index contrast at the bead/environment interface. First order modes are those modes with a single intensity maximum in radial direction, which is confined to the sphere/ambient interface. For further details of the mode assignment, we refer to the literature [4,9].

As we have shown in recent work on the application of WGM sensors to refractive index sensing [8], Equations 4 can be implemented into a numerical algorithm and used for simultaneous fitting of the measured WGM positions for bead radius *R* and refractive index contrast *n*. In the following, we will apply the same procedures for spectra fitting and subsequent numerical evaluation as presented in detail in said article, so that we refrain from a repetition here.

## 3. Results and Discussion

**Figure 2.**
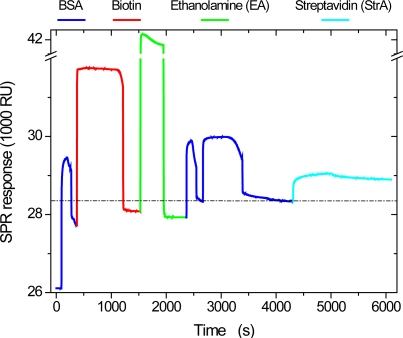
Surface plasmon resonance (SPR) response to a sequence of biomolecules adsorbed onto a polyelectrolyte-modified gold surface.

The sequence of biochemical reactions applied for exploration of the *in-situ* response of low-Q WGM sensors to specific biotin-streptavidin binding was first studied by SPR. At the beginning of each experiment, the surface of the SPR Au chip used was first functionalized with a carboxylated thiol, then two double layers of polyelectrolytes (PEs) were adsorbed to simulate the outer surface of the WGM sensors, which were also coated with two PE double layers after dye-doping (*cf.,* Section 4). Then, the surface was exposed to a sequence of treatment steps as shown in Figure 2, which displays the corresponding SPR response in resonance units as given by the instrument. First, a monolayer of BSA was deposited onto the outer PE layer, followed by exposure to an EDC/NHS activated biotin solution, its deactivation by ethanolamine, another BSA deposition, and finally streptavidin binding. In this sequence, the activated biotin couples supposedly to amino functionalities of the BSA via peptide bond formation, the second BSA adsorption is used to block non-specific adsorption sites potentially created by the activation/deactivation treatment of the surface, and finally, the streptavidin binds specifically to the biotinylated BSA. In control experiments applying the same sequence of treatment steps but lacking the biotin in the EDC/NHS solution, it was found that non-specific streptavidin adsorption is low (not shown). As can be seen in the Figure, upon injection of the different solutions, SPR exhibits a strong bulk effect, *i.e.*, the signal changes simply due to the difference in the refractive indices between analyte solution and running buffer. The adsorption can therefore only be quantified after termination of the injection, when the surface is once more exposed to the running buffer, which results in an increase of the baseline value in case of successful adsorption. The latter is a direct measure for the mass density of deposited material. As a rule of thumb, typically an increase by 1,000 RU corresponds to 1 ng/mm^2^ deposited protein.

**Figure 3.**
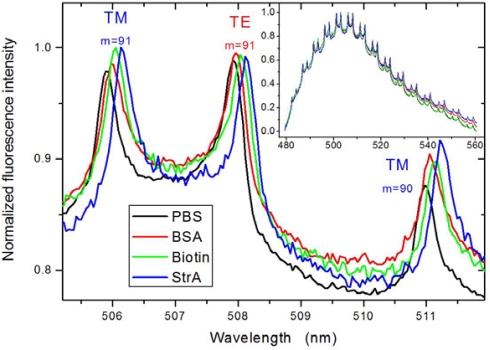
WGM spectra obtained from a single surface-adsorbed fluorescent polystyrene bead with a nominal diameter of 10 μm in dependence of the same sequence of biomolecules adsorbed onto the particle surface as used in the SPR study; the inset shows the overview over the entire emission wavelength range of the fluorescent dye applied, while the main figure displays a close-up of the most intense modes. The latter are labeled according to their respective polarization (TM/TE) and mode number *m*. It should be noted that under the given conditions, only 1st order modes are observable. PBS—phosphate buffered saline, BSA—bovine serum albumin, StrA—streptavidin.

The same sequence of surface treatments was then applied to the WGM sensor. Figure 3 displays its response to BSA adsorption, biotin coupling, and finally streptavidin binding, all obtained in running buffer after termination of the respective injection. The sequence shown in Figure 3 was recorded using a 600 L/mm grating, which limits the optical resolution, however, gives a good overview over the spectral evolution over the entire emission range of the fluorescent dye (for acquisition of the data used in the quantitative evaluation below, a 2,400 L/mm grating was applied to increase the optical resolution and thus the precision to which the WGM position can be determined. This was feasible because only few pairs of modes (we used typically three) are required for the evaluation). As shown previously, the WGM spectra obtained from fluorescently doped PS beads of about 10 μm in diameter immersed into an aqueous environment exhibit only *q* = 1 order excitations, which can be well described by Equations 4. The modes show up in pairs of TM and TE modes of same mode number, whereby *λ_TM_* < *λ_TE_*, and thus can be easily distinguished.

Upon biomolecular adsorption, the modes show a clearly observable red shift as expected from Section 2 and illustrated in Figure 1. For quantification of these shifts, the individual modes were first fitted via Voigt profiles to obtain their exact positions (cf. Table 1), which then could be further evaluated along the strategies discussed in Section 2.

The most vital question was if the application of the Airy approximations (Equations 4), which in prior work had proven to be quite robust in view of simultaneous determination of geometrical bead radius and environmental refractive index *n_e_*, would yield any improvement over the basic ray optics model of Section 2.1. In the latter, since the environmental index does not enter Equation 1, wavelength shifts *Δλ* can only be used for calculation of changes in bead size *ΔR* if measured in the same medium, similar to the procedure applied in the SPR study. An ultimate goal of *in-situ* biosensing on microscopic scale would be, however, to perform reference-free biosensing in any kind of environment, potentially even in live cells [16].

Therefore, the first evaluation of data followed the procedure of said prior study. The most important thing to mention is that also this time the sensors’ refractive index was first determined by fixing the environmental index to that of PBS, *n_PBS_* = 1.3338, which had been obtained by SPR. Subsequently, the bead index was fixed to thus obtained value (1.5427 ± 0.00250) and Equations 4 solved for bead radius and environmental index *n_e_*.

**Figure 4.**
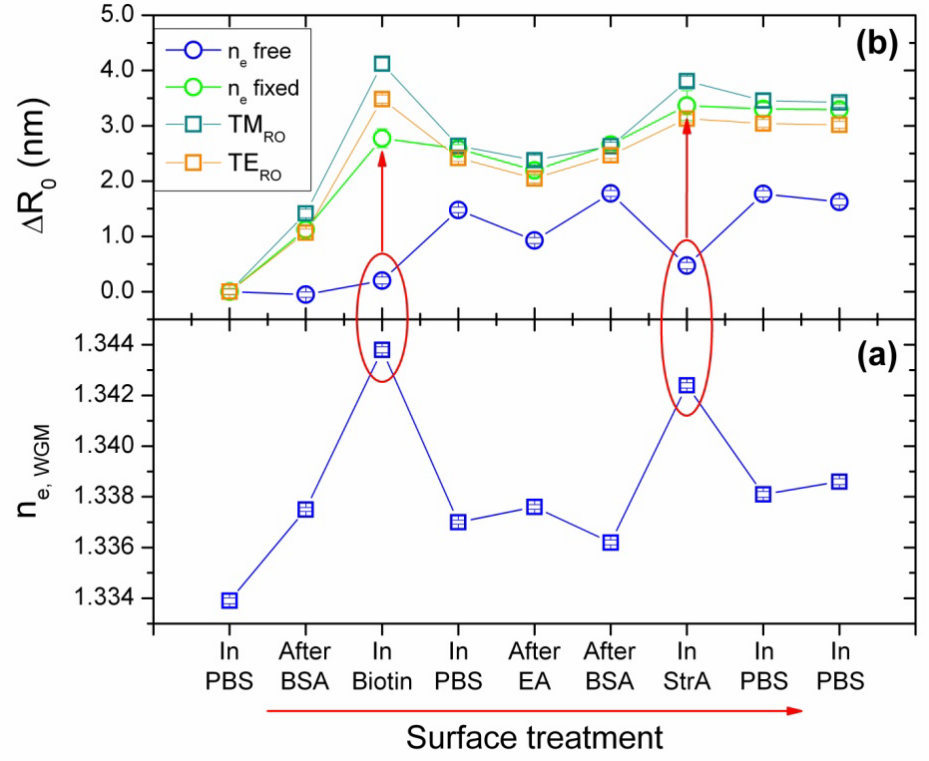
Results of the evaluation of WGM positions as determined after the indicated surface treatments; PBS—phosphate buffered saline, BSA—bovine serum Albumin, EA—ethanolamine hydrochloride, StrA—streptavidin, TM_RO_/TE_RO_—TM/TE mode determined by ray optics model, n_e_ free—simultaneous fitting for n_e_ and R by means of Airy model, n_e_ fixed—fitting only for R by means of Airy model, keeping n_e_ fixed to values obtained by SPR.

The results are shown in Figure 4. It should be noted that as detailed in the experimental section, most of the WGM measurements were performed in PBS buffer after termination of the respective treatment step indicated in the figure legend. Only the steps “In Biotin” and “In StrA” were performed in the NHS/EDC activated biotin and the streptavidin solutions, respectively. In SPR reference experiments, the refractive indices of PBS, biotin, and streptavidin solutions were determined to *n_PBS_* = 1.3339 ± 0.00080, *n_bio_* = 1.3374 ± 0.00080, and *n_StrA_* = 1.3339 ± 0.00080, so that *n_e_* in Figure 4a should deviate only for the “In Biotin” stage of the surface treatment from the PBS value.

In fact, except for the first measurement in PBS at the start of the experiment and the measurements in solutions other than PBS, the results for *n_e_* are rather constant, yielding *n_PBS_^WGM^* = 1.3375 ± 0.00084 on average, which is slightly above the SPR reference experiment, however, still within the respective errors. With *n_bio_^WGM^* = 1.3438 ± 0.00012, the index determined by the WGM sensor is slightly higher than that determined by SPR and lies outside the error. This trend is even more severe for the measurement in the streptavidin solution. The SPR does hardly show any bulk effect indicative of a solution index different from the PBS running buffer, while the WGM measurement gives an obvious increase to *n_StrA_^WGM^* = 1.3424 ± 0.00011. The reasons for this difference between SPR and WGM sensors is not clear, however, it gives a first indication that simultaneous fitting for n_e_ and R might not be feasible.

Our main interest in the present study is, however, if and how these differing results on *n_e_* may affect the quantification of the adsorbate layer. Figure 4b displays therefore the change in the sensor radius, *ΔR_0_ = R* − *R_0_*, from its initial value *R_0_* for R determined by simultaneous fitting of *n_e_* and *R* (blue curve). The evolution of *ΔR_0_* is obviously inversely correlated to that of *n_e_*. After BSA adsorption, which should give a significant increase in *ΔR_0_* due to the formation of a BSA monolayer, no increase is observable. In the biotin solution, the increase is small and only for the subsequent measurement in the PBS rinsing buffer, the effects of the prior treatments become observable. The same behavior can be observed for the subsequent treatment steps with that in the streptavidin solution as the most prominent, which makes clear that environmental refractive index *n_e_* and radius *R* mutually influence each other. This can be understood when inspecting the change of the WGM resonance positions in dependence of small changes of these two parameters, e.g., by calculating their partial derivatives from Equations 4. In the parameter range relevant here, the partial derivatives with respect to *n_e_* and *R*, respectively, are all positive and have a similar magnitude (for details, *cf.,* Appendix). Thus, both effects contribute to the observed change in the resonance positions to similar extent. This explains the complementary behavior of *n_e_* and *ΔR* in Figure 4. Why the fitting procedure yields an overestimation of *n_e_* at the cost of *ΔR*, however, is presently not clear and needs further investigation.

The situation changes when we fix *n_e_* to the respective values obtained by SPR for PBS, biotin, and streptavidin solutions and then use Equations 4 for determination of *R* only. The corresponding radius increase is also plotted in Figure 4b for comparison with the former results. The evolution of Δ*R_0_* (green curve) appears reasonable this time and—as shown in Figure 6—is also in good quantitative agreement with the SPR results. The radius increases clearly after the first treatment steps, *i.e.*, BSA adsorption and biotin coupling, then decreases slightly during PBS rinsing and ethanolamine activation, which may be understood as materials loss typically also observed by SPR. The second BSA adsorption yields only a small increase in the sensor radius, which corresponds to the presence of only few surface defects in the initially adsorbed BSA layer after biotin coupling and NHS/EDC deactivation. Specific streptavidin coupling, finally, yields a further increase, which is then stable in two subsequent PBS rinsing steps as can be expected from with high affinity specifically bound molecules.

Thus, by fixing *n_e_* to its expected value, the radius increase can be used for quantification of the adsorption layer. As marked in Figure 4b by the red circles and arrows, this effect is most prominent for the measurements in the biotin and streptavidin solutions, respectively. This is a somewhat discouraging result because it means that for the time being a reference-free simultaneous determination of *n_e_* and *ΔR_0_* as the most important parameters for *in-situ* WGM biosensing cannot be achieved. It wonders instead, if not even the simple ray optics model can be similarly applied for determination of *ΔR_0_*. If so, it would be easier applicable than the Airy model, because the solutions can be calculated analytically via equations 1 and 2.

To check on the performance of the ray optics model for determination of the increase in *R*, the peak positions obtained (Table 1) were also evaluated by means of equations 1 and 2, whereby different results were achieved for TM and TE modes, depending on which mode spacing was evaluated (between two neighboring TM or TE modes, respectively). The results for *ΔR_0_^TM^* and *ΔR_0_^TE^* are plotted together with the results of the Airy approximations in Figure 4b. Except for cases where *n_e_* differs from the PBS value, the agreement between ray optics model and Airy approximations with fixed *n_e_* is surprisingly good, with a deviation of typically 8%. Only in the case of the biotin solution with its higher index, the ray optics model overestimates the increase in *R*.

**Figure 5.**
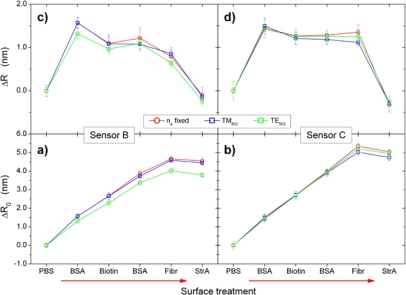
Same evaluation as shown in Figure 4b for two other sensor beads exposed to a series of surface treatment steps similar to the one shown in Figure 4. In contrast to the latter, the sensors were exposed to fibrinogen (Fibr) before injection of streptavidin; *ΔR = R_i_* − *R_j_*, where *R_i_* and *R_j_* are sensor bead radii obtained from two subsequent treatment steps, is the incremental radius increase and *ΔR_0_ = R_i_* − *R_0_*, where *R_0_* is the initial sensor bead radius, is the total radius increase, respectively.

That this good agreement is not just accidental was tested by evaluation of a total of six different data sets. As an example, Figure 5 displays the results for the changes in the radii obtained by the two models for two different sensor beads used in a control experiment. This time, after the BSA passivation step, the sensors were exposed to fibrinogen before incubation with the streptavidin solution, thereby suppressing specific binding of the latter molecule to biotin sites. Besides the total radius changes, *ΔR_0_* = *R* − *R_0_*, calculated as deviations from the initial sensor bead radius *R_0_*, also the incremental radius increases, *ΔR* = *R_i_* − *R_j_*, where *R_i_* and *R_j_* are the sensor bead radii obtained for two subsequent treatment steps, are shown. These incremental values *ΔR* are particularly important for the calculation of the mass density *σ* adsorbed in the respective treatment step according to Equation 3. While we observed as a trend that the ray optics result for the TM mode *ΔR_0_^TM^* was typically a better match to that of the Airy model *ΔR_0_*, Figure 5a/c show that even in such case the incremental radius changes *ΔR^TM^* and *ΔR^TE^* both may match satisfactorily the results of the Airy simulation *ΔR* and thus both may be used for the determination of adsorbed mass densities according to Equation 3. An evaluation of TE modes may have the advantage that the mode positions can be determined more precisely and under more severe conditions, such as low index contrasts, due to their typically smaller bandwidths as compared to their TM counterparts.

**Figure 6.**
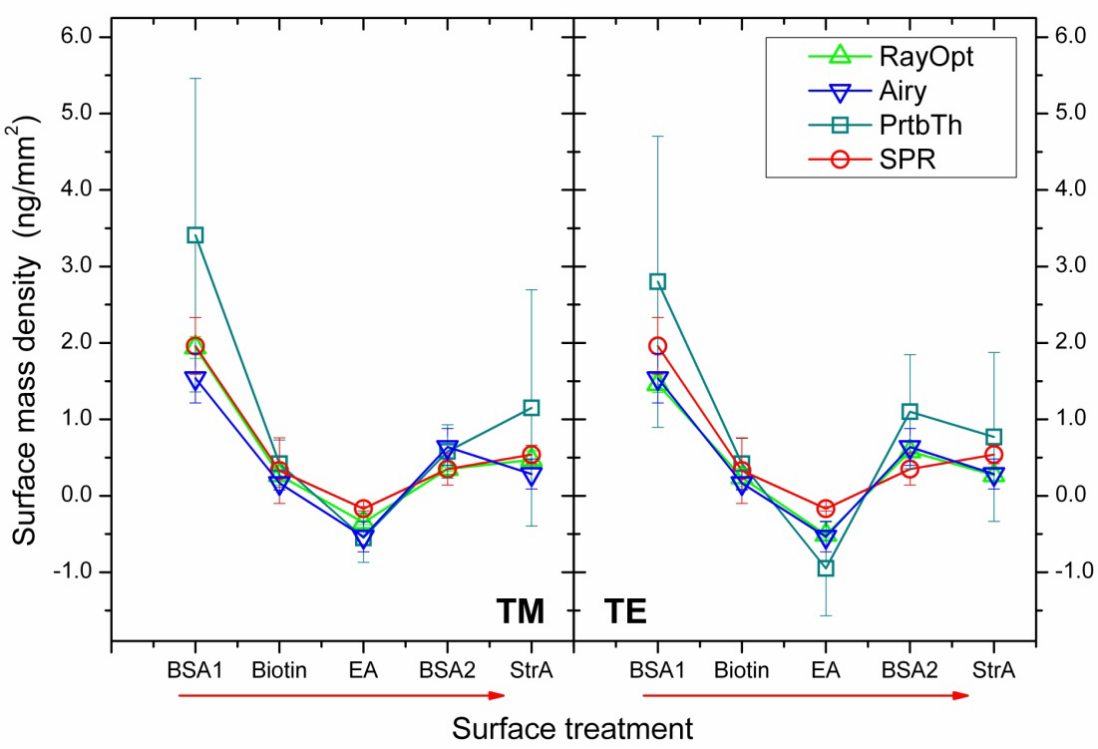
Surface mass densities adsorbed in the different steps of the biomolecular adsorption experiment as determined by SPR and the WGM sensor. For evaluation of the WGM sensor data (*cf.,* Table 1), three different models were applied: RayOpt—ray optics model, Airy—Airy model, PrtbTh—perturbation theory.

Nevertheless, for the data set treated here (Figure 4 and Table 1), the mass density per treatment step as calculated from the *ΔR^TM^* values on basis of Equation 3 are the best match to those of the SPR reference experiment. Figure 6 compares the mass densities obtained from the ray optics and the Airy models by exploiting *ΔR^TM^*, *ΔR^TE^*, and *ΔR*, respectively, with the results of the SPR reference experiment (*cf.,* Figure 2). While the agreement between the values is basically satisfying and particularly is within the respective experimental errors, the results based on evaluation of *ΔR^TM^* yield in fact excellent agreement with the SPR data. While this perfect match might be somewhat accidental, it should be kept in mind that the TM modes are typically more sensitive to changes in the sensor bead’s environment because of the presence of radial electric field components and therefore might provide the more sensitive transducer mechanism. Surprisingly, the Airy model is closer to the *ΔR^TE^* results despite the fact that the absolute radius increases, *ΔR_0_*, were closer to those of *ΔR^TM^* (*cf.,* Figure 4b). The reason here is probably that an absolute offset between *ΔR^TM^ and ΔR^TE^* is canceled out in the calculation of the incremental size changes *ΔR* and therefore does not necessarily influence the quality of the results.

To provide a broader view on reliability and applicability of the results obtained, Figure 6 also contains the results for the adsorbed mass densities based on first order perturbation theory as derived by Teraoka and Arnold [11]. For calculation of these values, we applied equations 29, 31, and 32 on page 1384 of said article, which describe thin-layer adsorption as is expectedly the case here. Surprisingly, the perturbation theory (“PrtbTh” in Figure 6) gives the most significant deviations from the SPR reference results and is mostly overestimating the mass density. This means actually that the perturbation theoretical approach underestimates the expected peak shifts per adsorbed mass unity, *i.e.*, that it predicts lower sensitivity of the WGM sensor. A sensitivity of low-Q WGM sensors higher than expected had already been found in some articles focusing on refractive index sensing [7,8] and ex-situ biosensing applications [9], indicating that perturbation theory does not fully describe the transducer mechanism in the case of small sensor bead dimensions in the range of few microns. The reason for this discrepancy is most likely that the perturbation theoretical approach of Teraoka and Arnold does not exploit the closed resonator condition and thus does not account for the wavelength shift caused by the change of the resonator size upon biomolecular adsorption. Instead, as can be seen, e.g., from Equation 8 of ref. [11], only the change in the refractive index is included and thus, the model does not differ essentially from those describing open-loop evanescent field sensors. While for high-Q WGM resonators with sizes in the sub-millimeter regime, *i.e.,* with diameters of about 100 μm and above, the change in resonator size upon adsorption of a biomolecular adlayer of few nanometers in thickness is negligible [17], it seems that for low-Q sensors with dimensions of some micrometers such omission is no longer possible.

## 4. Experimental Section

### 4.1. Materials

Polystyrene (PS) microspheres with a nominal diameter of 10 μm (n = 1.54 – 1.55 after dye doping [8]) were purchased from Polysciences, Inc., Warrington, PA, USA; poly(allylamine hydrochloride) (PAH), MW∼15,000 Da, poly(sodium 4 styrenesulfonate) (PSS), MW∼70,000 Da, 11-mercaptohexadecanoic acid (MHA), and xylene, p.a. grade, were received from Sigma-Aldrich K. K., Tokyo, Japan; glycerol, >99%, was obtained from Wako Pure Chemical Industr., Ltd., Osaka, Japan, Coumarin 6 laser grade (C6G) dye from MP Biomedicals, Solon, OH, USA, and polydimethylsiloxane (Sylgard 184; PDMS) from Dow Corning Co., Midland, MI, USA; all chemicals were used as received. Microscopy cover slips, 32 × 24 × 0.17 mm^3^, were obtained from Matsunami Glass Industr., Ltd., Osaka, Japan.

Streptavidin (StrA) and bovine serum albumin (BSA) were purchased from Thermo Scientific (Rockford, MD, USA). The BSA was received as 10% solution in PBS and diluted to 1% with PBS buffer before use. *N*-hydroxysuccinimide (NHS), 1-ethyl-3-[3-dimethylaminopropyl] carbodiimide hydrochloride (EDC), and ethanolamine hydrochloride (EA), 1 M, were obtained from Biacore K. K., Tokyo, Japan, as a part of the amine coupling kit. Phosphate buffered saline (PBS) was received in the form of tablets from MP Biomedicals and dissolved in deionized (DI) water yielding a pH of 7.2. The gold-coated glass chips used for the SPR measurements were obtained from Biacore K. K. (Tokyo, Japan) as a part of the Au SIA kit. DI water was produced with a Milli-Q system from Millipore, S.A., Molsheim, France.

### 4.2. Methods

The preparation of the sensor beads, the optical set-up utilized for sensor bead operation, and the settings for WGM spectrum acquisition are described elsewhere [8]. In the following, the procedures of biomolecule adsorption, the refractive index calibration of the SPR instrument, and the peak fitting routine applied to the WGM spectra are briefly summarized.

#### 4.2.1. Biomolecular Surface Treatment

The gold chips used for the SPR reference experiments were first functionalized with a carboxylated thiol (MHA) to provide a negatively charged surface onto which in subsequent steps two double layers of PAH/PSS were adsorbed (PAH: 1 mg/mL, PSS: 1.5 mg/mL; both in 0.5 M NaCl solution) to yield the same outer surface coating as that of the sensor beads [8].

The adsorption of BSA, biotin, and streptavidin onto the PE coated gold chips was then monitored *in-situ* by means of a Biacore *X* SPR system (Biacore K. K.), where PBS solution at pH 7.2 was used as the running buffer at a constant flow rate of 20 μL/min. The SPR response of the following consecutive pulses was monitored: (1) BSA (1%, in PBS); (2) biotin (1 mg/mL) in NHS+EDC mixture; (3) ethanolamine; (4) BSA injected a second time to ensure passivation of any available non-specific binding sites on surface; (5) streptavidin (1 mg/mL).

The experiment was repeated thoroughly to validate the results. In a first stage, only the formation of the PE layers was studied, followed by BSA adsorption. Since these experiments were performed in a broader study over several months, we obtained about 30 experimental results for both, WGM and SPR sensors. On this basis, the subsequent steps of biofunctionalization, *i.e.,* biotin coupling and streptavidin binding, were only performed when the BSA adsorption onto a PSS-terminated surface had been found to be successful with values in the range of 2000 RU for SPR and about Δλ = 0.15 nm for the WGM sensor corresponding to a monolayer of BSA.

The WGM sensor experiment was performed analogously. C6G-doped PS microspheres were first coated with two double layers of (PAH/PSS) and then drop-coated on PAH-coated microscopy cover slips. To ensure fixation of the beads even at high fluid viscosity, surface and deposited beads were coated with two more double layers of PAH/PSS. Then, the glass substrate was attached to a microfluidic flow cell made of PDMS bearing a rectangular flow channel of 15 × 2 × 0.1 mm^3^ in size.

Thus prepared sensor beads were then exposed to the same sequence of biomolecular solutions as described above, thereby applying the same flow velocities as used for the SPR experiments. SPR and WGM sensor experiments were performed in parallel, thereby using materials taken from the same aliquots to minimize any preparative errors. The running buffer was PBS as in the case of SPR. In contrast to SPR, however, the WGM response was occasionally also monitored during the exposure of the WGM sensors to a biomolecular solution, such as shown in Figure 4 for the measurements “In Biotin” and “In StrA”. In each experiment, a number of microspheres were traced to check on the reliability of the WGM shifts.

#### 4.2.2. Determination of Refractive Indices

The refractive indices of the different solutions used in the biomolecular adsorption experiments were obtained from the SPR sensorgrams by quantifying the bulk effect upon injection of the respective solution from the sharp drop in the signal at the end of the injection. These values were then converted into refractive index values according to the equation:

(5) $$n_{e}(\Delta \mathrm{RU})=(1.3311\pm 8.0\;\;10^{-4})+(0.0100\pm 2.0\;10^{-4})\;x\;\;\Delta \mathrm{RU}\;\;x\;\;10^{-4},$$

where *ΔRU* represents the change in SPR resonance units according to the respective bulk effect. This relation had been obtained before in a calibration experiment for the SPR system applying DI water/glycerol mixtures of different ratios (*cf.,* also Foley *et al*. [8,18]).

#### 4.2.3. WGM Spectra Evaluation

The evaluation of the WGM spectra proceeded in two steps. First, each spectrum was fitted by a number of Voigt profiles to yield the wavelength positions of the different modes observable. Then, these mode positions were used to calculate bead sizes, and in the case of the Airy model, also refractive indices of bead and ambient. The bead indices were calculated from the spectra in PBS buffer assuming *n_PBS_* = 1.33388, which had been determined before by SPR reference experiments according to the procedure outlined above. To keep consistency in the results, such obtained bead indices were also used for the ray optics model and perturbation theory. Errors were calculated by Gaussian error propagation based on the errors in the determination of the peak positions as given by the peak fitting routine (peak fitting module of origin 7.5Pro, OriginLab Co., Northampton, MA, USA). The errors of the results obtained by the Airy model were determined as described in prior work [8], which provides also a detailed analysis of instrumental and methodological errors.

## 5. Conclusions

Low-Q WGM sensors were applied to non-specific and specific biomolecular adsorption studies in terms of the well-established biotin-streptavidin model system. The results were compared with those obtained by SPR on the same sequence of surface treatment steps to demonstrate that quantitative evaluation of the obtained transducer signals is possible in a very similar fashion to that of SPR. This quantification in terms of surface mass densities adsorbed on the sensor surface is performed in two steps. First, from the wavelength shift upon (bio-)molecular adsorption, an effective change in the sensor size *ΔR* is calculated. Then, from this effective increase and the known mass density of the adsorbate, the surface mass density can be directly obtained from Equation 3.

For the conversion of the WGM wavelength shifts *Δλ* into an effective size increase *ΔR* of the sensor three different theoretical routines were tested. First, an elementary ray optics model was applied, then analytical Airy approximations to the full WGM wave solutions were used to numerically simulate the peak positions, thereby extracting information about sensor bead radii and environmental indices simultaneously. This procedure had proven successful in a recent application of low-Q WGM sensors to refractive index sensing [8]. However, in the present study we found that the accuracy of this method is not good enough to determine thin adsorption layers with sufficient precision when simultaneously fitting for the environmental index. Thus, reference-free remote biosensing in an arbitrary environment remains a challenge for the time being. This intricacy can be circumvented by measuring WGM spectra before and after a distinct treatment step in the same medium, e.g., the running buffer of the experiment. In this case, however, also the simple ray optics model gives reliable results, thereby lifting the need for time-consuming data fitting. Thus, for practical applications, such as the development of small and versatile optical sensors, a very simple relation between wavelength shift and effective bead size increase can be exploited for rapid data analysis and potentially real-time monitoring similar to present state SPR systems.

In addition to these two models, also a perturbation theoretical approach as recently proposed by Teraoka and Arnold [11] has been applied to our data. We found, however, that this description, which has been developed in view of sensor signal quantification of high-Q WGM sensors with sizes of several tens to some hundreds of micrometers, does not describe the WGM wavelength shift of low-Q WGM sensors well. The reason for this deviation is most likely related to the omission of the size increase of the sensor with the formation of the adsorption layer, which is still a reasonable practice at the size scale of high-Q sensors, but obviously is needed to describe the response of the low-Q sensors studied here. While admittedly also the other two models applied do not describe the situation of a sensor bead bearing an adsorption layer properly, since they neglect the difference in the refractive indices between sensor bead and adsorbate, this omission seems to be less crucial, thus indicating that we have entered a new physical regime here. It should be noted that in the present manuscript, we restricted the models applied to the evaluation of the WGM shifts to simple-sphere models. Future work will have to investigate if more complex models, such as the core-shell model of Aden and Kerker [19], will yield any advantage over the performance determined here.

Most importantly, we found reasonable agreement in the quantitative results obtained with the low-Q WGM sensor and the SPR reference, respectively. This is an encouraging result since the SPR device applied was a macroscopic system with a sensing area of about 800 times that of the microscopic WGM sensor bead. The latter, with its diameter of only 10 micrometers, therefore points a way for reliable label-free biosensing at a precision similar to that of SPR, however, on smaller scale and particularly with less effort. SPR imaging systems also promise sample analysis in the size regime of few micrometers, however, then require precise imaging of the sensor chip interface. In the case of low-Q WGM sensors, the demands on the excitation and detection optics in terms of acceptance angles and robustness of the opto-mechanics are significantly lower, since the crucial resonator condition is defined by the sensor bead itself and not by its periphery. It should be noted, however, that the present study was limited to a single biomolecular system, which is known to be very reliable and easily applicable. Thus, the quantitative agreement between SPR and WGM sensors found in this particular case might still be somewhat accidental and requires further studies applying more complex systems, such as antibody/antigen reactions, for its validation. Also, the potential influence of differences in the flow geometry, *i.e.*, adsorption onto a plane surface in the case of SPR *vs.* adsorption onto a sphere in the case of the WGM sensor, on the sensor performance need to be addressed in more detail in future work.

In comparison with high-Q WGM sensors, the supersession of the need for evanescent field coupling and the larger number of simultaneously detected modes, which allows determination of sensor bead radii from the mode spacings, is advantageous in view of ease of use and data quantification. These are only some of the reasons why the present approach seems to be promising, worth further exploration.

Altogether, we have shown that quantitative low-Q WGM biosensing can be successfully achieved at moderate levels of experimental and theoretical effort and thus encompass promising candidate systems for future low-cost biosensing applications on small scale.

## Figures and Tables

**Table 1.** WGM peak positions (in nm), their errors as resultant from the fitting routine (in nm), and the respective WGM quality factors for the WGM spectra of the different surface treatment steps of the biomolecular adsorption experiment as used for the quantitative evaluation shown in Figure 4. The modes are labeled according to their polarization (TM or TE) and numbered beginning with the modes with shortest wavelength.

**Surface Treatment**	**TM1**			**TE1**			**TM2**			**TE2**			**TM3**			**TE3**		

	**Position**	**Error**	**Q-Factor**	**Position**	**Error**	**Q-Factor**	**Position**	**Error**	**Q-Factor**	**Position**	**Error**	**Q-Factor**	**Position**	**Error**	**Q-Factor**	**Position**	**Error**	**Q-Factor**
**in PBS**	493.551	0.004	1,559	495.575	0.003	2,318	498.742	0.003	1,412	500.831	0.003	2,271	504.125	0.003	1063	506.263	0.002	2,273
**after BSA**	493.695	0.009	1,653	495.684	0.009	2,157	498.891	0.007	1,684	500.936	0.007	2,106	504.272	0.012	617	506.373	0.006	2,088
**in Biotin**	493.963	0.008	1,509	495.938	0.007	2,059	499.165	0.007	1,199	501.193	0.005	1,990	504.570	0.008	822	506.651	0.005	1,807
**in PBS**	493.811	0.010	1,387	495.819	0.012	1,796	499.013	0.008	1,999	501.074	0.009	1,792	504.401	0.017	523	506.519	0.008	2,100
**after EA**	493.825	0.010	1,357	495.780	0.008	1,898	498.988	0.006	1,501	501.031	0.007	1,855	504.381	0.010	707	506.483	0.006	2,316
**after BSA**	493.818	0.008	1,967	495.825	0.007	2,468	499.020	0.008	1,285	501.086	0.006	2,587	504.397	0.008	1,199	506.518	0.006	2,337
**in StrA**	493.943	0.009	1,733	495.897	0.009	1,391	499.140	0.010	824	501.151	0.007	1,766	504.526	0.009	925	506.588	0.006	1,922
**in PBS**	493.902	0.008	2,153	495.885	0.005	3,233	499.103	0.008	1,169	501.138	0.005	2,812	504.484	0.006	1,700	506.581	0.005	2,109
**in PBS**	493.892	0.011	1,760	495.885	0.008	2,428	499.104	0.011	1,056	501.132	0.006	2,782	504.480	0.009	1,302	506.577	0.006	2,507

## Appendix

Partial Derivatives of Equations 4:

$$\begin{array}{l} A_{\mathrm{TM}}\;\;:=\nu +1.8557\;\nu^{1/3}-\frac{1}{n\sqrt{n^{2}-1}}+1.0331\;\nu^{-1/3}-\frac{1.8557\;(n^{4}-\frac{2}{3})}{n^{3}{\left(n^{2}-1\right)}^{3/2}}\;\;\nu^{-2/3};\;\;\;\;\;n:=\frac{n_{s}}{n_{e}}\\ A_{\mathrm{TE}}\;\;:=\nu +1.8557\;\nu^{1/3}-\frac{n}{\sqrt{n^{2}-1}}+1.0331\;\nu^{-1/3}-\frac{0.6186\;n^{3}}{{\left(n^{2}-1\right)}^{3/2}}\;\;\nu^{-2/3}\\ B_{\mathrm{TM}}\;\;:=\frac{1}{n^{2}\sqrt{n^{2}-1}}+\frac{1}{{\left(n^{2}-1\right)}^{3/2}};\;\;\;\;C_{\mathrm{TM}}\;\;:=1.8557\;\nu^{-2/3}\left[\frac{3\;\;(n^{4}-\frac{2}{3})-{4n}^{4}}{n^{4}{\left(n^{2}-1\right)}^{3/2}}+\frac{3\;\;(n^{4}-\frac{2}{3})}{n^{2}{\left(n^{2}-1\right)}^{5/2}}\right]\\ B_{\mathrm{TE}}\;\;:=\frac{-1}{\sqrt{n^{2}-1}}+\frac{n^{2}}{{\left(n^{2}-1\right)}^{3/2}};\;\;\;\;C_{\mathrm{TE}}\;\;:=0.6186\;\nu^{-2/3}\left[\frac{-3\;\;n^{2}}{{\left(n^{2}-1\right)}^{3/2}}+\frac{3\;\;n^{4}}{{\left(n^{2}-1\right)}^{5/2}}\right] \end{array}$$

With these definitions, the partial derivatives of *λ*_TM_ and *λ*_TE_ with respect to *n_s_*, *n_e_*, and *R* according to Equations 4 read as follows:

$$\begin{array}{l} \frac{{\partial \lambda }_{\mathrm{TM}}}{\partial R}=2\;\pi \;n_{s}\;/\;A_{\mathrm{TM}};\;\;\frac{{\partial \lambda }_{\mathrm{TM}}}{{\partial n}_{s}}=2\;\pi \;R\;(1-n(B_{\mathrm{TM}}+C_{\mathrm{TM}})\;/\;A_{\mathrm{TM}})\;/\;A_{\mathrm{TM}};\;\;\frac{{\partial \lambda }_{\mathrm{TM}}}{{\partial n}_{e}}=2\;\pi \;R\;n^{2}(B_{\mathrm{TM}}+C_{\mathrm{TM}})\;/\;A_{\mathrm{TM}}^{2}\\ \frac{{\partial \lambda }_{\mathrm{TE}}}{\partial R}=2\;\pi \;n_{s}\;/\;A_{\mathrm{TE}};\;\;\frac{{\partial \lambda }_{\mathrm{TE}}}{{\partial n}_{s}}=2\;\pi \;R\;(1-n(B_{\mathrm{TE}}+C_{\mathrm{TE}})\;/\;A_{\mathrm{TE}})\;/\;A_{\mathrm{TE}};\;\;\frac{{\partial \lambda }_{\mathrm{TE}}}{{\partial n}_{e}}=2\;\pi \;R\;n^{2}(B_{\mathrm{TE}}+C_{\mathrm{TE}})\;/\;A_{\mathrm{TE}}^{2} \end{array}$$

As an example for the values of these derivatives in the present work, we set as follows:

*R* = 5000 *nm; ν* = 91.5; *n_s_* = 1.54; *n_e_* =1.35; *ΔR* = 3 *nm*; Δ*n_s_* = Δ*n_e_* = 0.01, thus yielding

$$\begin{array}{l} \frac{{\partial \lambda }_{\mathrm{TM}}}{\partial R}\;\;\Delta R=0.30\;\;\mathrm{nm}\;;\;\;\;\;\frac{{\partial \lambda }_{\mathrm{TM}}}{{\partial n}_{s}}\;\;{\Delta n}_{s}=2.8\;\;\mathrm{nm}\;;\;\;\;\;\frac{{\partial \lambda }_{\mathrm{TM}}}{{\partial n}_{e}}\;\;{\Delta n}_{e}=0.45\;\;\mathrm{nm}\;\\ \frac{{\partial \lambda }_{\mathrm{TE}}}{\partial R}\;\;\Delta R=0.30\;\;\mathrm{nm}\;;\;\;\;\;\frac{{\partial \lambda }_{\mathrm{TE}}}{{\partial n}_{s}}\;\;{\Delta n}_{s}=2.9\;\;\mathrm{nm}\;;\;\;\;\;\frac{{\partial \lambda }_{\mathrm{TE}}}{{\partial n}_{e}}\;\;{\Delta n}_{e}=0.36\;\;\mathrm{nm}\; \end{array}$$
